# Central Venous Catheter Placement Gone Awry: A Case Report of Right Internal Jugular Central Line Entering Subclavian Artery

**DOI:** 10.7759/cureus.31093

**Published:** 2022-11-04

**Authors:** Anita Subramanian, Nathan Richards

**Affiliations:** 1 Internal Medicine, Harnett Health System, Campbell University, Lillington, USA; 2 Medicine, Ross University School of Medicine, Bridgetown, BRB

**Keywords:** complication of central line, ultrasound guidance, cardiothoracic surgery, misplacement of cvc, central venous catheter (cvc)

## Abstract

While central venous access is necessary for a variety of situations including inadequate peripheral venous access, medication administration, hemodynamic monitoring, vasopressor administration, and hemodialysis, complications during the insertion process are not uncommon. In the United States, in both critically ill medical patients and surgical patients, millions of central venous catheters are inserted yearly. Complications occurring during or immediately following insertion include cardiac, pulmonary, and vascular injuries as well as issues with catheter placement. This case report describes a rare malposition of the central venous cannula into the subclavian artery. Few case reports of accidental subclavian artery catheterization have been published following internal jugular vein insertion. While arterial puncture is a well-recognized complication, accidental subclavian artery catheterization is even rarer than carotid artery cannulation. In the literature review, only two documented case reports for reference were found. There are severe risks associated with arterial cannulation including atherosclerotic plaque dislodgement, stroke, hemothorax, pseudoaneurysm, arteriovenous fistula formation, and death. This case follows a 78-year-old man who was brought in by emergency medical services (EMS) minimally responsive with hemodynamic instability - hypothermic, hypotensive, and tachycardic. The emergent decision was made to proceed with central venous catheter placement in the emergency department and placement was initially confirmed with radiologic evidence. Over the admission course, the patient had improvement in hemodynamic instability with minimal change in mental status, however, the need for further testing revealed the central line that was previously functioning without difficulty was arterial. Imaging demonstrated catheter traversed the internal jugular vein and inserting into the right subclavian artery requiring emergent transfer for vascular and cardiothoracic surgery intervention. While a rare complication, this case, differing from previously documented reports due to the delay in discovery, exemplifies how further investigation may be warranted to confirm catheter placement prior to removal to reduce the risk of life-threatening situations.

## Introduction

Central venous access is a common procedure performed in the clinical setting and while often a medically necessary procedure, it is not without risks. Although advances in imaging, access techniques, and devices have assisted in reducing types of complications, considerable morbidity and mortality can still result from central venous access. There is a multitude of complications associated with central line placement and this causes a significant burden on healthcare systems due to increased costs, prolonged hospital stays, and decreased quality of life. The most associated risks include vascular injury, infection, and misplacement [1]. Early recognition of complications and appropriate management are critical in caring for these patients. Case reports of complications have led to increased awareness and improved quality of care [2,3]. This article provides a report of an unexpected, rare malposition of a catheter in the right internal jugular vein.

## Case presentation

**Figure 1:**
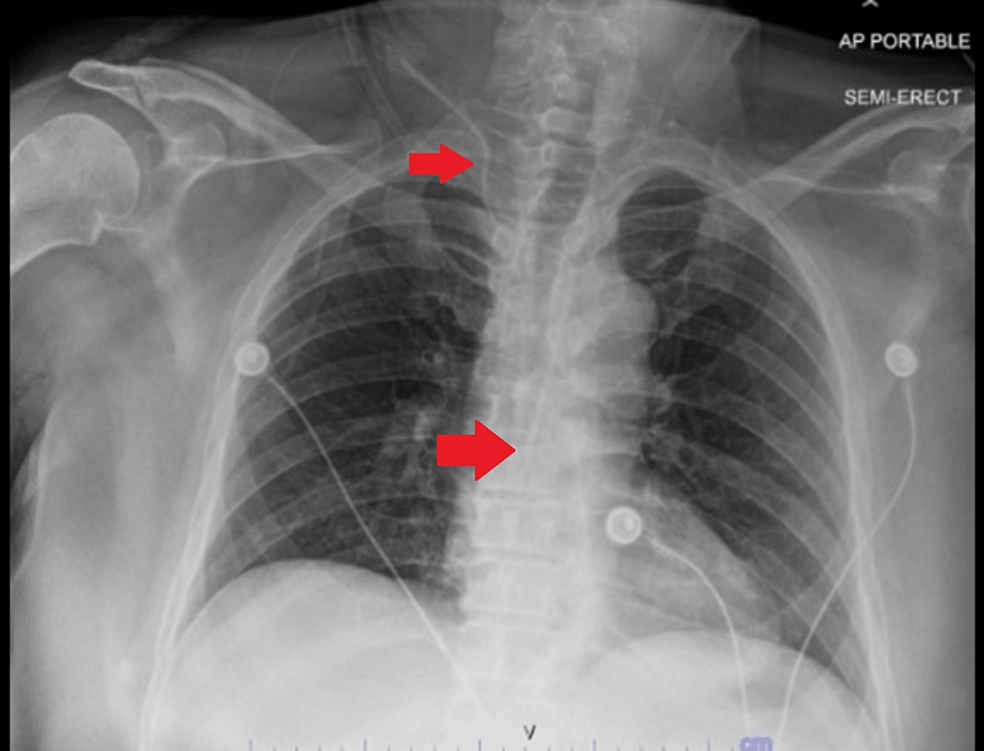
Chest AP radiograph confirming central venous catheter placement in the right internal jugular vein

A 78-year-old man, with a past medical history of type 2 diabetes, hyperthyroidism, and a ventral hernia was initially brought in by Emergency medical services after being found down for an unknown period of time at home. On the initial exam in the emergency department, he was minimally responsive, hypothermic, hypotensive, and tachycardic. Due to concerns about an inadequate response to fluid resuscitation and the need for vasopressor support the decision was made to proceed with the insertion of a central venous catheter for intravenous access. The emergency department physician identified the right internal jugular vein using ultrasound guidance. Under aseptic precautions after identification of the internal jugular, a vein decision was made to proceed with insertion. Per the emergency medicine physician due to hypovolemic status, insertion was difficult, and the wire was challenging to advance. Fortunately, the physician was able to advance the wire and cannulate the vessel after which the catheter functioned appropriately with blood aspiration and saline flushing. Intravenous fluids with normal saline infusion were infused through cannulated veins without difficulty. The central line position was initially confirmed using a chest radiograph as depicted in Figure 1.

**Figure 2:**
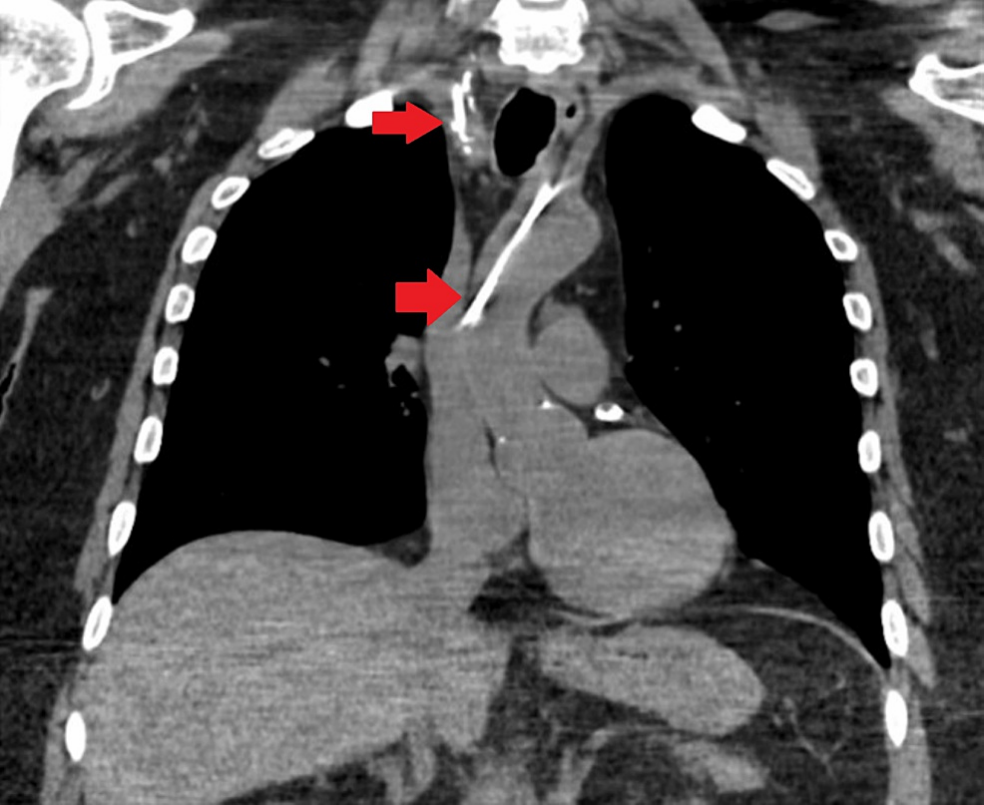
CT chest demonstrating catheter entering subclavian artery and tip terminating in the thoracic aorta

**Figure 3:**
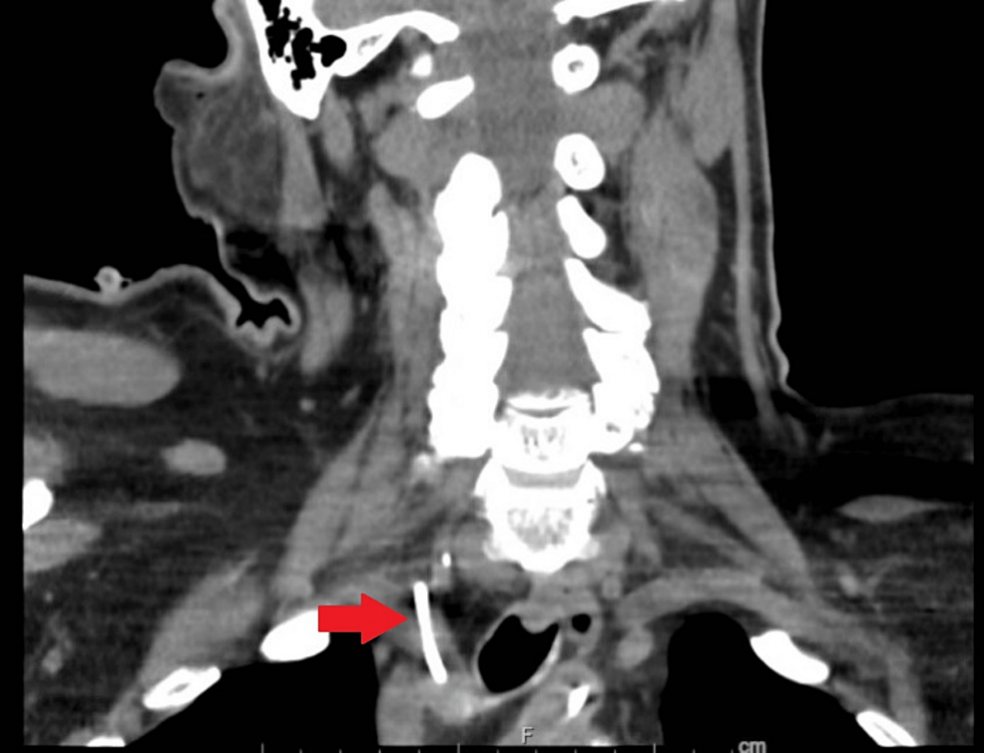
CT soft tissue neck demonstrating catheter traversing through the internal jugular vein to enter the subclavian artery

Over the hospital course, this patient received continued management for acute cerebrovascular accident and acute renal failure for which the central line was primarily used. On initial arrival, blood cultures were obtained due to hemodynamic instability and an unknown cause of mental status change. Due to persistent positive blood cultures and multiple embolic strokes seen on magnetic resonance imaging of the brain on arrival, the decision was made to proceed with a transesophageal echocardiogram. While in the pre-operative room, it was noted that there was some difficulty in drawing blood from the central line. A transducer was attached to the line and arterial pulsations were noted. Subsequently, an arterial blood gas was drawn which showed highly oxygenated blood. A repeat chest radiograph was performed demonstrating the central line tip terminating in the aorta. While blood draws were performed regularly over the hospital course, due to the abnormal placement, it is most likely that the different lumens drew from both arterial and venous sources delaying discovery. After one week of use, it is likely the lumen drawing from the venous source developed a clot and allowed for clearer detection during the procedure attempt. Tertiary care centers were contacted for a vascular surgery consultation and the need for transfer. Due to limited hospital availability, the patient was not immediately able to be transferred and it was recommended to obtain further imaging. A CT of the neck was performed shown in Figures 2, 3, which showed a mal-positioned right-sided central line traversing the anterior and posterior walls of the right internal jugular vein and subsequently inserting into the right subclavian artery with the catheter tip in the ascending thoracic aorta.

After reviewing the insertion process, it was determined that the initial imaging confirming placement was misread due to poor positioning of the patient due to the patient being rotated due to prolonged downtime. The decision was made at this time to proceed with the transfer to a tertiary care center for emergent evaluation and intervention by vascular and cardiothoracic surgery.

The patient was subsequently transferred to a tertiary care center and was taken to the operating room within twenty-four hours. The patient had extensive surgery performed with combined vascular and cardiothoracic surgery efforts. He underwent a sternotomy with catheter extraction, open subclavian artery repair, and right internal jugular vein ligation. The patient had a stable post-operative course and was transferred to a skilled nursing facility for rehabilitation.

## Discussion

While central venous catheter misplacements have been reported in the literature, subclavian artery catheterization is rarely seen. This case reports a misplacement of CVC in the right subclavian artery while attempting to secure an internal jugular vein central venous catheter. Before arrival and catheter insertion, the patient demonstrated hemodynamic instability requiring emergent placement of CVC. This case was unique in demonstrating the delayed discovery of the misplaced line and incorrectly confirmed placement on the chest radiograph. Furthermore, the patient improved hemodynamically over the admission course and the catheter did not demonstrate any difficulties with draw-back or flushing with medication administration and blood draws. Approximately 15% of catheters inserted have complications, 5%-19% have mechanical complications, 5%-26% have infectious complications and 2%-26% have thrombotic complications [4]. Although arterial puncture is a well-recognized complication occurring in 6.3%-9.4% of internal jugular vein cannulation attempts, accidental subclavian artery catheterization is even rarer than carotid artery cannulation which occurs in approximately 0.1%-0.5% of cases [4]. Typically, arterial cannulation is identified by bright red blood on a puncture or pulsatile flow; however, in cases of hypovolemia or hypotension, this is more difficult to identify on initial placement. The patient likely remained stable despite a misplaced line because CVC was primarily used only for intravenous fluid administration, and even with initial hemodynamic instability the patient never required vasopressor support. Additionally, arterial cannulation is normally identified by difficulty in administering IV fluids hence the delayed discovery in this case. A catheter inserted in the right subclavian artery typically lies above the clavicle on a chest radiograph but because this placement traversed the internal jugular prior to insertion in the subclavian artery it was not properly identified on the radiograph. Rapid identification of misplaced central lines is extremely important in preventing further complications.

Complications of catheter insertion

Complications of central venous access can often be avoided with proper sterile technique and with advancements in medical technology such as using ultrasound guidance to assist in placement. Risks associated with complications include morbidity, mortality, and increased healthcare costs.

Immediate complications

Cardiac complications such as arrhythmias are caused by guidewire contact with the right atrium. These are easily managed with the removal of the guidewire. Vascular complications are typically associated with the highest mortality and include arterial injury or bleeding, hematoma, or venous injury. These have been reduced due to the advancement of placement techniques and the use of ultrasound guidance. However, various studies have shown the incidence of arterial puncture as high as 4.2% to 9.3% in central line placements [5]. Pulmonary complications include pneumothorax, tracheal injury, or air emboli. Reports have shown pneumothorax or pneumomediastinum occurs in about 1% of cases [1]. Catheter placement complications include entrapped or lost guidewire requiring surgical or fluoroscopic guidance removal.

Delayed complications

The most common delayed complications of central line placement include infection or dysfunction of the catheter. Infection most commonly occurs from organisms typically related to normal skin flora such as Staphylococcus aureus and Staphylococcus epidermidis but can be severe resulting in sepsis, shock, and ultimately death. Dysfunction of a catheter can result from fibrin sheath formation blocking the opening of the catheter or venous thrombosis. Serious complications with leaving an improperly functioning catheter in place can cause arrhythmias, endocarditis, limb edema, or paresthesia [6].

## Conclusions

While central venous access is necessary for a variety of situations including inadequate peripheral venous access, medication administration, hemodynamic monitoring, vasopressor administration, or hemodialysis, complications during the insertion process are not uncommon. These adverse events are both critical to the patient and expensive to resolve. In the United States alone millions of central lines are inserted yearly. Approximately one-sixth of catheters inserted have difficulties ranging from mechanical, infectious and thrombotic issues. Complications occurring during or immediately following insertion include cardiac, pulmonary, and vascular injuries as well as issues with catheter placement. Internal jugular cannulation is often the first choice of approach for central venous access. The most common complications associated with internal jugular cannulation include pneumothorax, bleeding, and infection.

This case report describes a rare malposition of the central venous cannula into the subclavian artery. Few case reports of accidental subclavian artery catheterization have been published following internal jugular vein insertion. Severe and even deadly risks are associated with arterial cannulation. Although the procedure in this instance was performed by a highly trained physician, this report highlights one of the various ways in which complications during placement can arise. It demonstrated how even with insertion utilizing ultrasound guidance and confirmation by chest radiograph, images can be misinterpreted due to poor visualization and lead to incorrect confirmation of placement. While a rare complication, this case exemplifies how further investigation and multi-disciplinary discussion with radiology and the medical team may be warranted to confirm catheter placement prior to removal to reduce the risk of life-threatening situations such as acute hemorrhage.
